# Automated IoT Device Identification Based on Full Packet Information Using Real-Time Network Traffic

**DOI:** 10.3390/s21082660

**Published:** 2021-04-10

**Authors:** Narges Yousefnezhad, Avleen Malhi, Kary Främling

**Affiliations:** 1 Department of Computer Science, Aalto University, Tietotekniikantalo, Konemiehentie 2, 02150 Espoo, Finland; narges.yousefnezhad@aalto.fi (N.Y.); amalhi@bournemouth.ac.uk (A.M.); 2 Department of Computing and Informatics, Bournemouth University, Fern Barrow, Poole, Dorest BH12 5BB, UK; 3 Department of Computing Science, Umeå University, Mit-Huset, 901 87 Umeå, Sweden

**Keywords:** device identification, IoT Security, device profiling, real-time traffic, machine learning

## Abstract

In an Internet of Things (IoT) environment, a large volume of potentially confidential data might be leaked from sensors installed everywhere. To ensure the authenticity of such sensitive data, it is important to initially verify the source of data and its identity. Practically, IoT device identification is the primary step toward a secure IoT system. An appropriate device identification approach can counteract malicious activities such as sending false data that trigger irreparable security issues in vital or emergency situations. Recent research indicates that primary identity metrics such as Internet Protocol (IP) or Media Access Control (MAC) addresses are insufficient due to their instability or easy accessibility. Thus, to identify an IoT device, analysis of the header information of packets by the sensors is of imperative consideration. This paper proposes a combination of sensor measurement and statistical feature sets in addition to a header feature set using a classification-based device identification framework. Various machine Learning algorithms have been adopted to identify different combinations of these feature sets to provide enhanced security in IoT devices. The proposed method has been evaluated through normal and under-attack circumstances by collecting real-time data from IoT devices connected in a lab setting to show the system robustness.

## 1. Introduction

We are witnessing the dawn of the Internet of Things (IoT) era, providing interconnection between the physical and the digital worlds with a remarkable impact on every aspect of our lives. Correspondingly, IoT has been paving its way in multiple sectors, recently including household and industrial solutions. The number of IoT devices can be predicted to reach up to 500 devices per household by 2022 [1]. In IoT-based smart city environments, an enormous number of physical devices are employed throughout the city, which are highly accessible and consequently, making their physical security of paramount importance. Such issues related to weak physical security as easy device disassembling, access of device data by malicious means, and removable storage media are considered major security threats [2]. Henceforth, despite the various benefits imparted by them in terms of flexibility and usability, these also entail a multitude of security concerns and attacks [3]. A foremost consideration in attack prevention for IoT devices is isolating such devices with restrictive communications to other devices through a gateway. Apparently, instead of device isolation, proper IoT device identification is a better approach for network administration considering security risks [4].

Additionally, the provision of a secure communication channel is another challenge apart from physical security. Presently, secure communication is provided by public and private key pairs or certificate installation on these devices, which helps provide the identity of the device. Again, there are limitations to using these certificates; for instance, if these certificates are accessed by an unauthorized entity, it will allow identity theft, allowing the transmission of false data to other devices in the network. It will impact the decision-making process because decisions are usually made on the aggregated data from different devices and a single falsification of data will affect the entire system. As a countermeasure, hardware-based and software-based approaches can be applied. The Root of Trust (RoT) is an example of hardware-based solutions that can be equipped by using either hardware like a chip called the Trusted Platform Module (TPM) or using software like Trusted Execution Environment (TEE). Using RoT, which is embedded on an IoT device, the authenticity of other certificates can be verified by the root, which is chained to those certificates [5].

**Figure 1:**
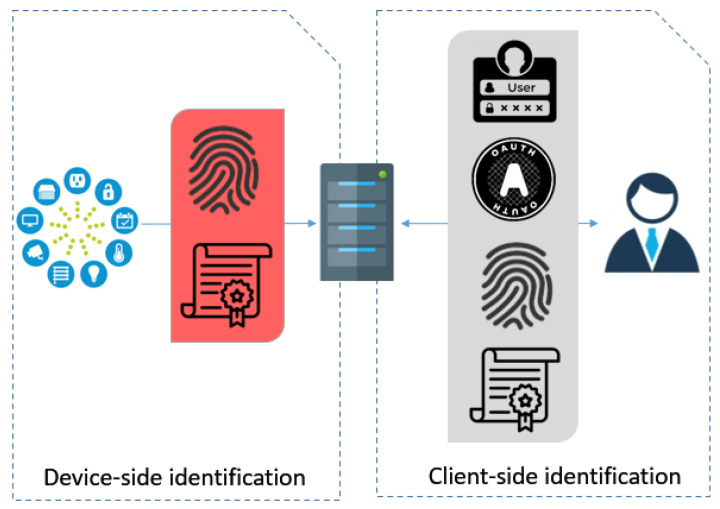
Identification in Internet of Things (IoT).

Apart from hardware-based approaches to countermeasure these types of identity thefts, the system should be capable enough to identify these threats. The identity claimed by the user is validated and proved by employing authentication techniques which include certificates, local user/password setting, or OAuth server connection (as shown in client-side identification of Figure 1). This problem has been resolved by using Facebook authentication with OAuth2 and an Access Control List (ACL) approach, where access rules are specified using a tree-based information structure [6].

In contrast, there are very few options for verifying the device identity as shown in device-side identification of Figure 1. The main challenge on the device-side is to authenticate the origin of the received messages on the server to detect identity thefts. One solution is using a certificate, which can easily be spoofed. Device fingerprinting can be considered a more optimal solution in which the process of identification of connected devices can be automated by network administrators [7]. Similar to fingerprinting, device profiling can be an approach for continuous device identification by monitoring behavioral features.

**Paper Contribution**: This work addresses the challenge of IoT device identification within a network by analyzing and classifying network traffic data for device identification from arriving packets with high accuracy by taking machine learning (ML) approaches. We solve this problem by presenting an identification approach based on sensor measurements, statistics, and header information for device behavior or device profiling by monitoring the data packets coming from smart devices to protect the server from receiving and spreading false data. Each device’s behavior is defined by its features, which are characterized by seven profiling models specified in the proposed framework. We adopt ML methods to learn unique features of each IoT device without expert supervision and evaluate them using network performance and algorithms’ accuracy. For proof-of-concept, we implement a demonstrator system for the IoT system comprising of temperature and humidity sensors integrated through open standards called Open Messaging Interface (O-MI) and Open Data Format (O-DF). The present paper is an extended version of our paper, presented at the INDIN conference [8]. This paper significantly expands the feature sets and develops realistic results by running a real use-case, collecting real data, and evaluating the system through various attack scenarios.

## 2. Literature Review

As there is a need for multiple users and devices to authenticate each other using trustable services, it is imperative that identity authentication be managed in IoT networks. The device identification idea for handling data privacy was first coined by Sarma and Girao [9] in 2009, however, most of the identity management systems based on Service Oriented Architecture (SOA) in IoT such as Shibboleth, Card-Space, and Liberty Alliance rarely considered the identity of devices in the framework [10]. To identify the device identity, the security module can either employ its identity or its specific features. Recently, various types of features in communication networks have been adopted by researchers for device identifications. The most common features lately applied could be location based on Global Positioning System (GPS) and Wi-Fi [11,12], the familiarity of devices derived from Bluetooth, time [12], identity (MAC—Media Access Control, IP—Internet Protocol, or RFID—Radio-Frequency Identification tag) [12], unique hardware-specific characteristics such as clock skew [13,14,15], device fingerprinting using MAC and properties of packets received from a device such as address and port of client and server [16], inter-arrival time [17], padding, packet size, and destination IP counter [18]. During attack detection, real-time and environmental factors also need to be considered to avoid false alarms [19].

Header information has been adopted extensively for device identification methods in the literature. A device identification technique has been proposed for the identification of the device model and type based on header information’s similarity calculation and this method is built for factory-used devices and network cameras by relying on general communication information [20]. Another method employs device fingerprinting for authentication and identification purposes by training an ML method based on extracted features from the network traffic for the detection of similar types of devices [21]. An automated classification system was also developed for device characteristics known as System Identifier (SysID) [4]. They applied Genetic Algorithms (GA) to distinguish the most unique features and various ML methods to classify the device type. ML algorithms were used in another approach on network traffic data for device identification of the devices connected in an IoT network [22]. The labeled data were employed for training and validating the classifier where labeled data were collected from nine unique IoT devices, smartphones, and personal computers. A multi-stage meta classifier was trained using supervised learning in the first stage to distinguish the IoT and non-IoT devices generating traffic and in the second stage, a unique class has been associated with each IoT device.

In some use-cases, the non-white list (not allowed to be used within any premises of organization) devices for trustworthy IoT device types need to be detected. For this purpose, a supervised ML algorithm, Random Forest (RF) was adopted to train extracted features from network traffic data with an objective to correctly identify the types of IoT devices from the white list [23]. A multi-stage classification algorithm was developed based on the network activity, demonstrating its ability to identify particular IoT devices [24]. A device identification approach was proposed where packet sequences from high-level network-flow traffic data were analyzed using supervised ML techniques to extract distinct flow-based features to create device fingerprinting [25]. The proposed approach was able to automatically identify device types in the white list and the individual device instances in the IoT network. Furthermore, a system security model was also designed to enable rules enforcement to constrain IoT device communications according to their privileges. This helps in suspicious device identification with abnormal behavior restricting their communication to avoid further monitoring. A similar ML approach based on sent and received packet streams was proposed to recognize connected IoT device types in an experimental smart home network and the designed model helps describe IoT device network behaviors [26]. Another system named AuDI (Autonomous Device Identification) [27] was proposed by analyzing the device-related network communications to identify the device type in an IoT network traffic. An unsupervised learning algorithm was used for modeling periodic IoT devices’ communication traffic for identification. Hence, after the initial learning phase, AuDI’s operation is fully automatic for the identification of previously unfamiliar devices and device types in any operational mode or device lifecycle stage.

Table 1 represents a summary of state-of-the-art from various perspectives. Most of the current identification methods focus on identifying the types of IoT devices from the predefined list of legitimate devices while our method verifies identities of the devices by making profiling models for each of them. The proposed method in this paper is considered a traffic analysis method with certain differences. Since payloads mostly are encrypted in traffic analysis, payload information is not operational in constructing the fingerprinting. However, in our implementation, due to the new messaging standards (O-MI/O-DF), the unencrypted payload data are accessible on the server. Therefore, we adopt the payload data for device identification alongside header information and generated statistical features. The idea of using payload data or sensor measurements for device identification is initially proposed by [8]. This identification framework employs only payload data and some extracted statistical features from a public dataset without taking into consideration any attack scenarios. However, in the current paper, by collecting data from a real use-case, header information can also be extracted to be combined with payload data, which creates a variety set of diverse features. In addition, the paper accomplishes the experimental results by evaluating the classification results under various attack situations.

## 3. Device Identification Framework

**Figure 2:**
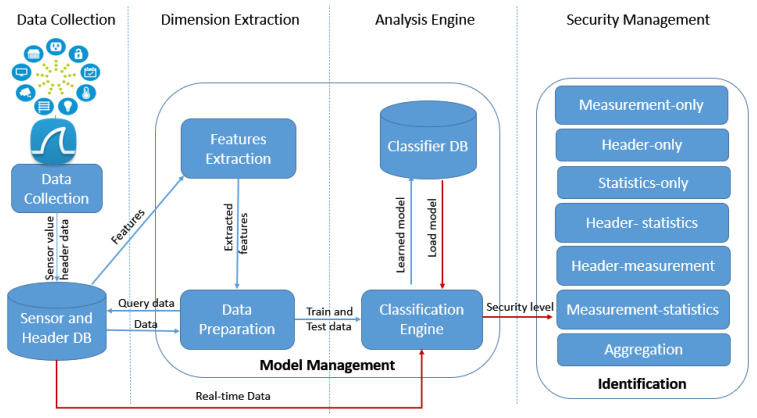
IoT device identification framework.

Figure 2 presents a high-level overview of the framework, which makes device identity decisions by performing automatic classification of IoT devices. Such classification works based on sensor measurements, traffic data, and a classifier model. This system overall encompasses four layers: data collection, dimension extraction, analysis engine, and security management. The system also includes two key modules, namely, *Model Management* and *Security Management*. *Model Management* will be responsible for detecting the most adequate features and investigating their weight or importance through ML methods. Once the dimensions have been extracted and the ML model of the current device is identified, *Security Management* decides on the security options, whether it is authenticated or not, and provides the enforcement support. Additionally, two databases (DBs) are also accessible to manage the data. The first DB, *Sensor and Header DB* consists of the sensor measurements, header information, and dimension names. Once the required dimensions have been loaded from the first DB, the value of each dimension will be elicited from the observation and the learned model will be stored in *Classifier DB*.

This is a data-driven framework obtained from sensor devices via GPS (Global Positioning System), Wi-Fi, and Bluetooth at the beginning of the *Data Collection* layer. Simultaneously, a packet analyzer such as Pyshark captures the traffic features extracted from packet headers. Then, the *Features Extraction* module applies all the features for calculating the feature vector to describe the present observation based on the value of the extracted features. Subsequently, a data query will be sent to *Sensor and Header DB* by the *Data Preparation* module for finding the necessary sensor and header information. During the device’s learning phase, the sensor data and extracted features are trained to build the model for the device using a training and testing set. The *Classifier DB* stores the trained model from the *Classification Engine*.

Once the learning phase is completed, it is time for the prediction phase (red lines in Figure 2), as the next step in model processing. After extracting the features from the new input, the features and the classifier models loaded from the *Classifier DB* will be employed by the *Classification Engine* to classify new observations. As the classification result is prepared, the engine will assign a security level (e.g., binary value) to the classifier and forward it to the *Security Management* layer, which considers the security level during the verification of device identity. The *Identification* module verifies the identity and labels the available feature set of current observations as malicious or legitimate. The feature set in the last module can be composed of header features, sensor measurement, some statistical features, or a combination of them all.

## 4. Enforcement

The implementation details including system methodology and steps, model creation and selection, and feature extraction are discussed as follows.

### 4.1. Methodology

**Figure 3:**
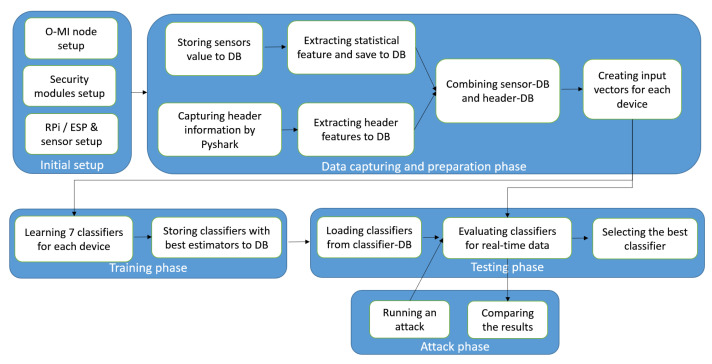
Implementation steps.

To describe the device identification framework, Figure 3 depicts a workflow of implementation steps. As seen in the figure, the first step in implementing the device identification framework is to set up the environmental modules including the O-MI node, security modules on top of the O-MI node, IoT gateways such as Raspberry Pi (RPi) and Electronic Stability Program (ESP), and sensors connected to IoT gateways. From thereon, all the modules will be operated over the O-MI server, which is implemented on a virtual machine at Aalto University. All these modules generate a concrete security module that is easy to plug-in on various servers, due to its modularity. On the other hand, running all the security modules in a single place convert it to a new product that can be reused on any other smart environment with minor patches.

Once all the required modules are running, in the *Data capturing and preparation* step, the sensor measurements can be stored in the sensor-DB on the O-MI server. Simultaneously, header information related to HTTP messages arriving from the sensors can be captured and extracted to the header-DB. *Python* has been selected as the core programming language of this phase and the remaining phases. Thus, to allow Python packet capturing and packet parsing, a Python wrapper called *Pyshark* has been employed which uses Wireshark dissectors (or tshark).

Alongside the sensor measurements and header features, few time-based features are also calculated based on the date-time attribute measured by sensors. Such features generate the third category called statistical features, which will be stored in sensor-DB. Therefore, for each sensor data sent to the server, three categories of features (sensor measurements, header features, and statistical features) can be incorporated in seven combinations as input vectors for the *Training phase*. Accordingly, seven classifiers are defined for each gateway device with their best estimators. These classifiers will be stored in classifier-DB and later during the *Testing phase*, they will be fetched from the same DB. Once the classifiers are loaded in the *Testing phase*, seven input vectors similar to the training vector but containing testing data are created and the real-time data are evaluated with previously learned classifiers. The best classifier is selected based on the evaluation results. Finally, to investigate the effect of attacks on the evaluation results and if the best classifier can efficiently find the attack, some attacks will be implemented and the performance results will be compared.

### 4.2. Model Creation and Selection

Since it is a classification problem, first we need to collect data for both classes (normal and abnormal) and train the model according to these data using ML algorithms, which are chosen based on the data linearity or non-linearity. Then, we can use this model to classify the new data. We use classification since it is more accurate than unsupervised models. For this purpose, we make a unique classifier model for each device that serves as the device profile. We employ the One-Vs-Rest classifier also called the One-Vs-All classifier, which is a multi-class classifier training one classifier per class. As a result, N classifiers will be created for N classes [25]. To achieve this, any $flow_{x}$ (i.e., a sequence of packets) captured on the server will be labeled either as 1 or 0 in *n* profiles (n = number of devices). For instance, to create the profile for $device_{i}$ ($profile_{i},i=1,2,\ldots ,n$), the label for $flow_{x}$ equals 1 if such flow arrives from device *i* and equals 0 if it arrives from other devices j≠i. In other words, $flow_{x}$ will be labeled as 1 in $profile_{i}$, and as 0 in $profile_{j}\left(j\neq i\right)$, as seen in Equation (1). As a result, *n* device profiles are available for learning the model. Then after finishing the device profiling, the features in profiles (including sensor measurements, statistical features, and header information) will be divided to make seven possible models as inputs to ML algorithms. The models are Statistic-only, Measurement-only, Header-only, Statistic-Measurement, Statistic-Header, Measurement-Header, and Statistic-Measurement-Header or Aggregation.

(1) $$profile_{i}=\left\{\begin{array}{cc} 1 & \mathrm{if}\;x=i\;(x\;\mathrm{is}\;\mathrm{id}\;\mathrm{of}\;\mathrm{arriving}\;\mathrm{flow})\\ 0 & \mathrm{if}\;x=j\;\left(j\neq i\right)\; \end{array}\right.$$

Once the models are created, the ML algorithm will acquire the models for each device and learn or load the classifier whether it is in the training or testing phase. To implement the ML algorithms, Scikit-learn Library parameters are applied with their best estimators.

### 4.3. Feature Extraction

In this paper, considering six sensor sets and IoT gateways, the real-time sensor data and their header packet information are collected on the virtual machine and stored in separate datasets including header-DB and sensor-DB. Packet headers were captured using Pyshark, and the nominated header features have been stored in header-DB. It is imperative that the verification of continuous identity considers the selected features. Once the sensor data are received by the server, the data and their behaviors are verified by comparison with the previous values present in the feature database.

All binary or integer features with zero variance such as IP version (value=4), IP header length (value=20), IP protocol (value=TCP), IP header checksum (value=2) were removed from header-DB. Besides, to avoid redundancy, we keep only one feature among highly correlated features. For instance, the Sequence number is stored between the Acknowledgment number and the Sequence number or the Next sequence number is chosen between the Segment length and the Next sequence number. Besides, sensor-DB consists of two sensor measurements (Temperature and humidity) and two statistical features (flow duration, inter-arrival time). The inter-arrival time is calculated for each packet according to the time interval between two consecutive packets (current and previous packets). Table 2 represents the list of features with their importance rate calculated through the RF algorithm. Features with a higher importance rate have a more significant impact on the algorithm results. As seen in the table, the sensor measurements and statistical features have a much higher impact compared to header-based features.

## 5. Adversary Model

As IoT devices are spreading around the world, cyber-attacks are becoming more sophisticated in multiple stages by coordinating various attacks from different places, thus requiring more complex defense mechanisms and vulnerability assessments. To properly assess the vulnerabilities, the network requires to face a simulated attack. In other words, a real synthetic attack scenario will occur in the network so that the potential breaches or vulnerabilities and the related defense mechanisms can be identified [28]. In this investigation of two general attack categories called physical attack and remote access attack, we simulate two attacks from these categories including object emulation attack and Botnet attack. However, only one set of results is presented in the paper, since both attacks have the same effect on the system, and as a result, they yield similar evaluation results. “we introduce two types of common attack scenarios in IoT environments, which can be detected by our device identification approach. We want to show that our model not only identifies the device identity but also can detect the attacks originating from the identity theft.”

### 5.1. Our Attack Model

#### 5.1.1. Physical Attack

**Figure 4:**
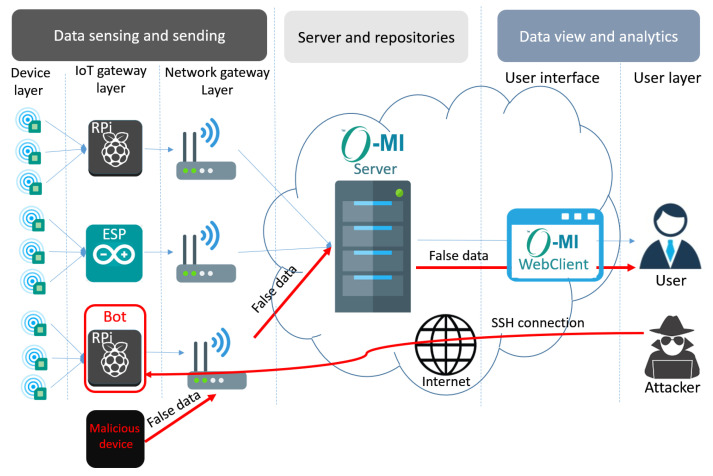
Object emulation attack and Botnet attack model in IoT.

A physical attack or *object emulation attack* [19] occurs when an extra device of the same identity as an authorized device is attached to the IoT network where this identity is used for falsification of the messages to convey an illusion as if the actual legitimate user is sending these messages. The device certificates are physically accessed from an authorized device by an attacker for installation on any other IoT device, by which the malicious device is granted access to send wrong data to the server (see the malicious device in Figure 4). These false data can be used by an attacker to trigger a false alarm (e.g., fire alarm) inducing a disaster. Hence, this type of physical security generally possesses two possible solutions [29]: network layer security control or barrier placing around the network. As barriers in open environments and large spaces, such as public places and smart campuses are impractical, the exploration of the network layer security control is a more feasible solution.

#### 5.1.2. Remote Access Attack

*Botnet attack* is the most popular remote access attack. The attacker remotely connects to an RPi (bot) through a Secure Shell (SSH) connection simply by knowing its login credential. Then, using this bot, the attacker can control the data sending process from the sensor to the server. From now on, whenever the user requests temperature and humidity data, the bot can forge the data passing the RPi. For instance, if the sensor measurement is 24 for temperature and 26 for humidity, the bot modifies the data to 10 and 10. Eventually, by receiving the false temperature and humidity, the user might turn on the heater or humidifier and triggers a critical incident in environments such as hospitals demanding careful consideration. Figure 4 displays the attack scenario.

## 6. Evaluation

In this section, initially, the testing scenario and performance metrics will be defined. Then, according to the introduced metrics, the classification results will be analyzed in various circumstances: the normal implementation of the network with no attack and the implementation while the attack running on the network.

### 6.1. Scenario Description

**Figure 5:**
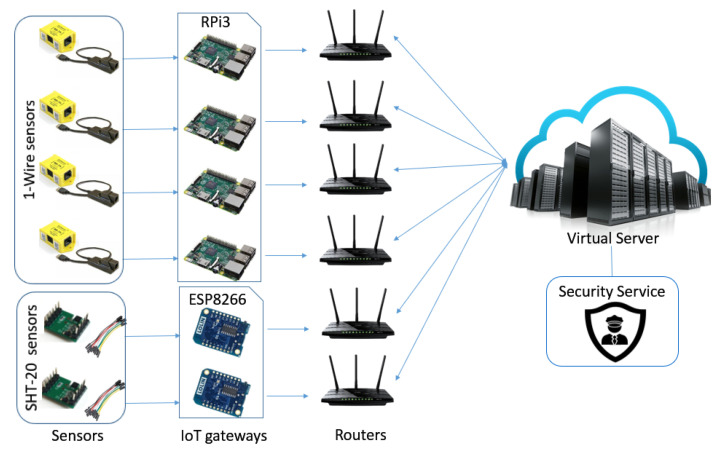
Experimental setup for data collection.

To evaluate our approach, a prototype system in a real environment (i.e., an office) has been established as shown in Figure 5. It generally contains six sensors, six IoT gateways, six wireless routers, a virtual server, and the security module running on the server. In such a prototype, two different temperature and humidity sensors including 1-Wire and SHT-20 are respectively connected to two IoT gateways Raspberry Pi 3 (RPi3) and ESP8266, which are all installed in an office in the Aalto ASIA (Adaptive Systems of Intelligent Agents) Lab. By connecting to the Internet through various wireless routers, IoT gateways forward the sensors’ data in tree-based O-DF ontology to the O-MI server. In other words, a wrapper is running over the IoT gateway which reads the sensor value, translates it to the O-DF, makes an O-MI Write request, and finally sends an HTTP POST request. The O-MI server then manages the device identification via running the security service.

Based upon three categories of features (measurement-based, header-based, and statistical), seven profiling methods are established: Measurement-only, Header-only, Statistic-only, Measurement-Header, Measurement-Statistic, Header-Statistic, and Aggregation. The Measurement-only, Header-only, and Statistic-only methods include only the sensor measurements (Temperature and Humidity), header features (first set of features in Table 2), and statistical features (flow duration, inter-arrival time), respectively. In the same vein, the Measurement-Header, Measurement-Statistic, and Header-Statistic methods contain a combination of two sets of features as defined in their names. Finally, the Aggregation method combines all three sets of features into one model.

### 6.2. Performance Metrics

Various performance metrics have been used for the evaluation of the efficiency of the proposed system, which are described below.

#### 6.2.1. Confusion Matrix

The exactness and reliability of a system are calculated through a confusion matrix which is also termed an error-matrix. This matrix promotes a clear idea of the system performance by analyzing the misclassification rate and accuracy.

- True Positive (TP): When the predicted and actual classes are identically at true class (1).
- True Negative (TN): When an element is predicted to be in False class (0) and it truly belongs in false class (0).
- False Positive (FP): When the system predicts an element to be in true class (1) but in actual it does not.
- False Negative (FN): When the system predicts that an element does not belong to a false class (0) but in actual it does.

#### 6.2.2. Accuracy

*Accuracy* refers to the total number of correct classifications performed by the network out of the total number of classifications made. As shown in Equation (2), accuracy is the rate of true predictions by all the true and false predictions combined.

(2) $$Accuracy=\frac{\left(TP+TN\right)}{\left(TP+FP+FN+TN\right)}$$

#### 6.2.3. Recall or Sensitivity

*Recall* is the rate of accurately predicted positives to genuine positives. Recall provides us with an idea about a model’s performance proportionate to false negatives.

(3) $$Recall=\frac{TP}{\left(TP+FN\right)}$$

#### 6.2.4. Precision

*Precision* is the rate of accurately predicted positives to all the predicted positives. Precision is about predicting frames correctly, whereas Recall is about predicting all the positive frames correctly [30]. So, to minimize false negatives, we must focus on enhancing Recall as high as possible with a decent and acceptable Precision value. The values of both Precision and Recall can be monitored by a single value performance metric called the F1-score.

(4) $$Precision=\frac{TP}{\left(TP+FP\right)}$$

#### 6.2.5. F-Score

To consider the role of both precision and recall, the *F*1*-score* is computed. As presented in Equation (5), the *F*1*-score* is simply the harmonic mean of precision and recall. In the case of unbalanced class distribution in the dataset, the *F*1*-score* is a more optimal evaluation metric than accuracy. A low value of the F1 score indicates a problem when either Precision or Recall has a low value. In that case, the *F*1*-score* is closer to the smaller value than the bigger value out of these two.

(5) $$F1-score=\frac{\left(2\times Precision\times Recall\right)}{\left(Precision+Recall\right)}$$

### 6.3. Classification Results under Normal Situation

During the training phase, data are collected for 25 h in Comma-Separated Values (CSV) format in which columns represent the nominated features and each row includes a list of features related to one arriving packet. Then, the seven profiling systems defined in Section 4.2, will be trained through three ML methods, namely, RF, Support Vector Machine (SVM), and Logistic Regression (LR). Henceforth, during the testing phase, once the data are collected for the next 5 h in CSV data format, the trained models will be adopted to verify the device identity by measuring the performance metrics. This process continuously occurs every five hours. Due to data collection being performed in 25 and 5 h, the ratio between the training and testing set will be 80–20, which is the most common proportion of training and testing data. The average values of performance metrics are calculated for six classifiers corresponding to each IoT device. The experimental results for three ML methods are represented in Table 3, Table 4, Table 5, Table 6, Table 7, Table 8 and Table 9.

Considering all the tables, it can be determined that SVM has the highest accuracy and F1-score compared to other ML methods in most of the profiling models. Moreover, although both SVM and LR generally perform fast, SVM has the fastest building time (in seconds) in our case scenario. Hence, we nominate SVM for attack analysis in the next step. Our data have two fundamental properties: (I) they include a relatively small level of sparsity, and (II) classes are not linearly separable. SVM can nicely handle binary classification tasks where the classes are not linearly separable while LR is unable to cope with such problems well. We think the reason why SVM outperforms RF is because of the first property of the data above. Although the sparsity level is not large, it can still negatively affect the performance of RF. Furthermore, SVM is intrinsically suitable for binary classification tasks, same as our classification task, while RF is suitable mostly for multi-class classification tasks. On the other hand, regarding the profiling system, the Measurement-only model reports the best results for all the ML methods. The next best models are measurement-statistic and measurement-header. Therefore, we can conclude that models including measurement features generally perform better compared to the rest of the profiling models.

### 6.4. Classification Results under Attack Situation

This section evaluates performance results through the attack scenario where the SVM method is employed for device identification. In other words, the best classifiers in the previous analysis under normal situation is employed in this section to detect attacks and the effect of attacks on the results is evaluated based on the FP rate. As a result, we can analyze whether the best profiling models and features in a normal situation are also able to efficiently detect the attack or other models and sets of features operate better under the attack situation. Two main attack scenarios are introduced. In the first scenario (Scenario 1), the attacker forges both sensor measurements including temperature and humidity while in the second scenario (Scenario 2), only one of them (temperature) will be modified by the attacker through one infected IoT gateway. The attacker could also send the forged data in different inter-arrival times (30, 50, 70, and 100), in which 100 s is the same as the original inter-arrival time of the legitimate device. Since the attack should be detected at the earliest opportunity, we evaluate the model performance over each scenario for every hour.

**Figure 6:**
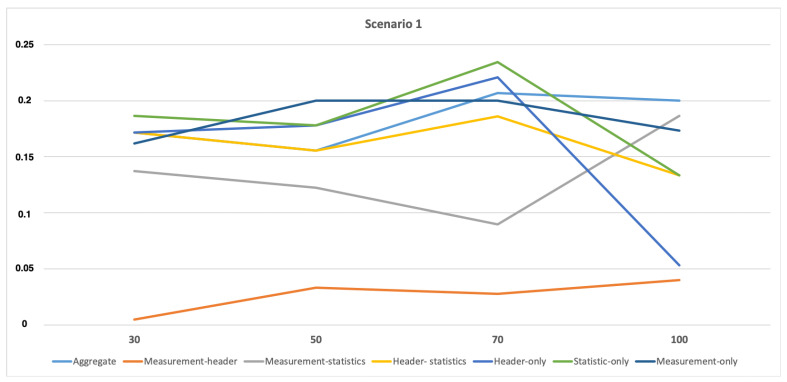
False Positive (FP) rate for attack scenario 1 for all the profiling models.

**Figure 7:**
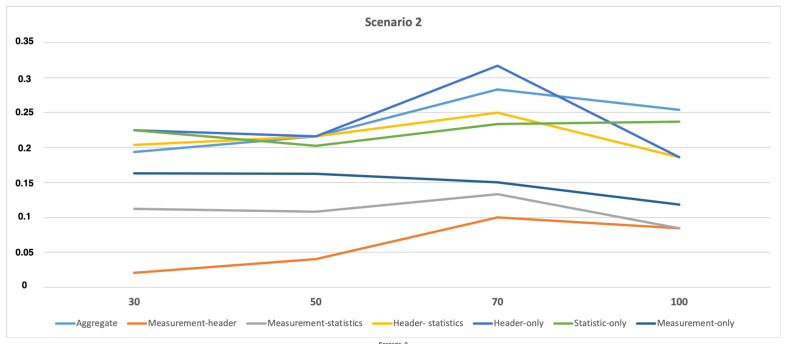
FP rate for attack scenario 2 for all the profiling models.

A FP rate is selected to analyze the performance of various profiling methods during attacks. The lower FP rate represents a higher performance for the profiling model. As seen in Figure 6 and Figure 7, the profiling methods with *measurement* features (i.e., Measurement-header and Measurement-statistics) have a minimum FP rate explaining that these can be employed for higher performance. In other words, the best profiling methods under normal circumstances can also make the best performance results during the attack scenario. It can also be explained with the help of Table 2, where measurement features have the highest importance but when they are combined with header features (which have the next highest feature importance) they even perform better, which is clear from the results presented in Figure 6 and Figure 7.

**Figure 8:**
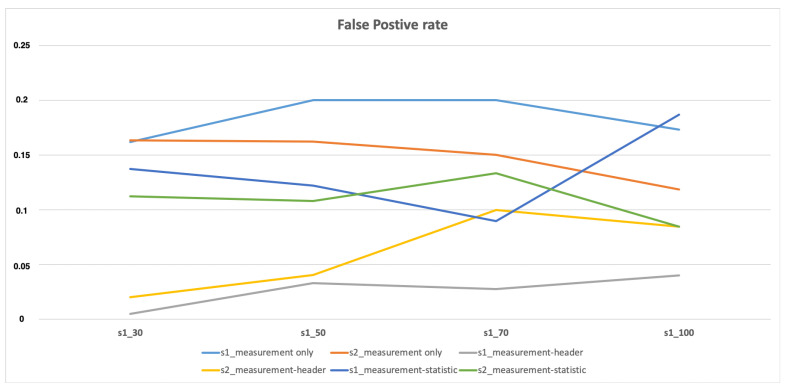
FP rate for attack scenario 1 (s1) and scenario 2 (s2).

As seen in Figure 8, the measurement-header has the lowest FP rate for both Scenario 1 and Scenario 2. At the same time, measurement-statistic has a slightly higher FP rate and finally measurement-only has the highest while comparing these three profiling methods. Hence, when measurement features are combined with other features especially header features, they perform on a higher level.

Since measurement features have a more profound effect on the results, they are selected and the performance metrics for the measurement-only and measurement-header are calculated using the SVM classifier and presented in Table 10. The values are calculated for the different inter-arrival times for both the attack scenarios. Further, the measurement header performs better than measurement only for all the inter-arrival times.

## 7. Conclusions and Future Work

After proposing an IoT device identification in our previous work, and presenting two profiling methods, we learned that this framework is fixable enough to run more variety of profiling methods. Accordingly, seven profiling models have been defined. First, three methods are based on a single data category (the sensor measurement-only, header-only, and the statistic-only). Second, three methods are based on double data categories (measurement-header, measurement-statistic, and header-statistic). Finally, the last type is the aggregation of all features provided by the first three methods. The performance of these models is evaluated by implementing a real IoT use-case. Our results show a significant accuracy improvement in the measurement-based models, especially the measurement-header model. Future work ought to be dedicated to increase the type and number of IoT devices and to collect real-time network data for a longer period of time. We also plan to include behavioral features for device identification.

## Figures and Tables

**Table 1:** Comparison of the proposed work with state-of-the-art techniques.

Pap.	Purpose	Identification Method	Considered Features	Implementation	Attack Status
[20]	To identify the device type and device model	Calculating the similarity of features	Communication features extracted from header	Network cameras and factory-used devices	No attack
[21]	To employ behavioral fingerprinting for identification and authentication	K-nearest-neighbors (K-NN), Decision Trees (DT), gradient boosting, and majority voting	Header feature and payload-based features	14 home IoT devices	No attack
[4]	To automatically classify the IoT devices using TCP/IP packets	ML algorithms (DT, K48, OneR, PART) to classify device type	GA to determine most unique features from network, transport, and application layer	a database from [18]	No attack
[22]	To identify IoT devices using ML algorithms on network traffic data	Two-stages classifier: I. distinguish IoT vs non-IoT II. determine device class	features from network, transport, and application layer + data from Alexa Rank and GeoIP	9 distinct IoT devices, and PCs and smartphones	No attack
[23]	To identify IoT device types from the white list	multi-class classifier using RF	Features from Transmission Control Protocol/Internet Protocol (TCP/IP) sessions	17 different IoT devices (9 device type) by different vendors	Based on local organizational security policies violations
[24]	To classify IoT devices using traffic characteristics	multi-stage ML: Stage-0. Naïve Bayes Stage-1. RF	statistical attributes: activity cycles, port number, signaling patterns, and cipher suites	a living lab with 28 IoT devices	User Datagram Protocol (UDP) reflection and TCP SYN attacks
[26]	To recognize IoT devices by analyzing the generated network traffic	RF, DT, Support Vector Machine (SVM), k-NN, Artificial Neural Network and Gaussian Naive Bayes	Size of first 10 pack sent/ received and interval times	experimental smart home network of 4 devices	No attack
[25]	To automatically identify white-listed device types	ML classifiers ( e.g., SVM and K-NN)	behavioural and flow-based features	31 off-the-shelf IoT device (27 device types)	Adversaries compromising devices on network
[27]	To identify device-type without human intervention	unsupervised learning method	4 types of features: periodic flaws, periodic accuracy, period duration, and period stability	a dataset comprising 33 typical commercial IoT devices	Spoofing device fingerprints
Our work	To identify the device using device profiling	ML methods (RF, SVM, and Logistic Regression (LR))	header information, sensor measurements, and statistical features	2 types of sensors in an office	physical and remote attacks (Object emulation and Botnet attack)

**Table 2:** List of features.

Type	Attribute	Header Sub-Attributes	Importance
Header (19)	Network layer (2)	‘length’ ’time_to_live’	0.0429 0.0071
	Transport layer (15)	‘source_port’ ‘stream_index’ ‘length’ ‘sequence_number’ ‘next_sequence_number’ ‘header_length’ ‘window_size_value’ ‘window_size’ ‘window_size_scalefactor’ ’options’ ‘analysis_initial_rtt’ ‘analysis_bytes_in_flight’ ‘analysis_push_bytes_sent’ ‘time_relative’ ‘time_delta’	0.0095 0.0034 0.0498 0.0288 0.0662 0.0069 0.011 0.0187 0.0061 0.0077 0.0017 0.0214 0.0341 0.0334 0.0037
	Application layer (1)	’content-length’	0.0655
	Packet length		0.0514
Measurements (2)	Temperature		0.2409
	Humidity		0.0597
Statistics (2)	Flow duration		0.1997
	Inter-arrival time		0.181

**Table 3:** Classification performance for aggregation.

	Accuracy	Recall	Precision	F_Score	Build Time
RF	81.36%	0.6666	0.5205	0.7989	12
SVM	86.20%	0.6734	0.5574	0.8134	2.96
LR	81.33%	0.8475	0.5888	0.8305	15.06

**Table 4:** Classification performance for Measurement_Header.

	Accuracy	Recall	Precision	F_Score	Build Time
RF	86.21%	0.4611	0.4799	0.8232	13.56
SVM	88.47%	0.5792	0.6949	0.8696	2.91
LR	85.41%	0.9247	0.6704	0.8633	17.75

**Table 5:** Classification performance for Measurement_statistic.

	Accuracy	Recall	Precision	F_Score	Build Time
RF	85.65%	0.4958	0.4236	0.8334	12.64
SVM	89.42%	0.7932	0.6664	0.8935	4.20
LR	91.79%	0.5909	0.7595	0.8518	3.88

**Table 6:** Classification performance for Header_statistic

	Accuracy	Recall	Precision	F_Score	Build Time
RF	81.82%	0.5101	0.3975	0.7871	12.20
SVM	81.47%	0.5984	0.4803	0.808	3.13
LR	78.45%	0.7540	0.5483	0.7959	16.38

**Table 7:** Classification performance for Measurement_only.

	Accuracy	Recall	Precision	F_Score	Build Time
RF	89.64%	0.5885	0.6786	0.8777	11.93
SVM	92.62%	0.6461	0.7911	0.9118	3.4
LR	89.66%	0.5263	0.5766	0.8609	2.46

**Table 8:** Classification performance for Header_only.

	Accuracy	Recall	Precision	F_Score	Build Time
RF	80.35%	0.3803	0.2884	0.7665	12.88
SVM	82.57%	0.4482	0.4106	0.7958	5.95
LR	80.14%	0.6440	0.4679	0.7922	19.75

**Table 9:** Classification performance for Statistic_only.

	Accuracy	Recall	Precision	F_Score	Build Time
RF	71.94%	0.2939	0.1719	0.6929	11.55
SVM	72.55%	0.2915	0.2288	0.6894	2.2
LR	75.99%	0.0448	0.05	0.7005	2.58

**Table 10:** Support Vector Machine (SVM) results for measurement-only and measurement-header during attack scenarios.

Scenarios	Inter-Arrival Time (s)	Accuracy	Recall	Precision	F_Score
Measurement-Only					
Scenario 1	30 50 70 100	80% 76.19% 74.12% 74.44%	0.4 0.4 0.2 0.2	0.2556 0.24 0.2 0.0667	0.815 0.7648 0.7592 0.7029
Scenario 2	30 50 70 100	79.09% 77.65% 75.71% 75.71%	0.2267 0.2 0.1 0.04	0.26 0.1333 0.2 0.2	0.8194 0.7892 0.7675 0.782
Measurement-Header					
Scenario 1	30 50 70 100	93.91% 89.52% 88.24% 0.9%	0.4 0.36 0.18 0.4	0.37 0.35 0.2 0.36	0.9131 0.8742 0.855 0.8804
Scenario 2	30 50 70 100	90.91% 88.24% 81.43% 84.29%	0.16 0.2 0.15 0.2	0.2 0.2 0.2 0.2	0.8884 0.862 0.8107 0.8331

## Data Availability

The data used to generate the plots and figures can be accessed by contacting the author at narges.yousefnezhad@aalto.fi.
